# Functional brain alterations in anorexia nervosa: a scoping review

**DOI:** 10.1186/s40337-016-0118-y

**Published:** 2016-11-28

**Authors:** Tone Seim Fuglset, Nils Inge Landrø, Deborah Lynn Reas, Øyvind Rø

**Affiliations:** 1 Regional Department for Eating Disorders, Division of Mental Health and Addiction, Oslo University Hospital, Ullevål, Oslo, Norway; 2 Department of Psychology, Clinical Neuroscience Research Group, Faculty of Social Sciences, University of Oslo, Oslo, Norway; 3 Department of Psychology, Faculty of Social Sciences, University of Oslo, Oslo, Norway; 4 Division of Mental Health and Addiction, Institute of Clinical Medicine, University of Oslo, Oslo, Norway

**Keywords:** Anorexia nervosa, Functional magnetic resonance imaging, Scoping review

## Abstract

**Background:**

Neuroimaging allows for the identification of brain abnormalities and alterations that are associated with anorexia nervosa (AN). We performed a scoping review to map out the extent and nature of recent research activity on functional magnetic resonance imaging (fMRI) in individuals diagnosed with, or recovered from, AN (AN-REC).

**Main text:**

A literature search of PubMed, Psychinfo and Embase was conducted using the search terms “anorexia nervosa” AND “functional magnetic resonance imaging.” We included fMRI studies that involved a comparison between individuals with AN or AN-REC and healthy controls published in English language between 2010 and 2015. A total of 49 papers were included, regardless of the experimental stimuli or paradigm.

**Conclusions:**

Findings varied considerably across studies, reflecting methodological differences in study design, such as sample differences and experimental paradigms. Collectively, studies published during the past five years suggest altered activation in regions related to the fronto-striato and the limbic circuits, which are theorized to have an important role in the pathophysiology of AN.

## Plain english summary

Anorexia nervosa (AN) is a severe mental disorder, and is characterized by self-induced starvation and excessive weight loss. The cause of the disorder is not known, however, it is assumed that altered activation in different parts of the brain could contribute to the symptoms and behavior seen in AN. A range of studies have been performed, to investigate brain activation in patients with AN, however the findings are varying. This could possibly be explained by differences in study designs, such as experimental conditions and sample size. We have collected the studies between 2010 and 2015, to map the nature of these studies. Based on gaps in the literature, we have made suggestions for future studies which are important to address to increase our understanding of AN. Furthermore, we have summarized the main findings from these studies. Although these findings are varying, they indicate altered brain activation in regions related to the fronto-striato and the limbic circuits, which have previously been theorized to have an important role in AN.

## Background

Anorexia nervosa (AN) is a complex mental disorder with an unknown etiology. There is a growing body of evidence indicating that risk for AN is genetically linked and that underlying neural networks may sustain the illness [1], similar to many other psychiatric illnesses [2]. Neuroimaging technology allows for investigating alterations in neural networks of the brain, and how these underlying neurobiological processes might contribute to the symptoms and behaviors seen in AN. However, to date, it is not known which regions and networks of the brain are involved in the etiology and pathophysiology of this illness. This paucity in knowledge has stimulated a rapidly growing literature base. An increasing number of functional magnetic resonance imaging (fMRI) studies have been published over the past few years geared toward the identification of brain regions and neural circuits potentially involved in AN. Neural responses to disorder-specific images related to body image or food, for example, have been investigated among patients with AN, and neural activation or alterations during the performance of such tasks has been documented in the literature [3, 4].

However, these studies often vary in terms of experimental paradigms and stimuli, posing challenges to the interpretation and synthesis of findings. Prior review papers have focused rather narrowly upon specific experimental stimuli or affected area, for example, the nature of limbic dysfunction in AN, with a specific focus on emotional and perceptual neural circuits [5]. Other reviews have focused on imaging studies investigating neural responses to food cues [3], processing of food, body and emotional stimuli [6], and body image distortion [4]. Friederich et al. [7] reviewed brain imaging literature related to the anxiety and pathological fear learning model of AN, as well as the impulsivity learning of binge eating in bulimia nervosa, while Wierenga et al. [8] focused on recent studies that showed altered sensitivity to reward and punishment in eating disorders. Frank & Kaye [9] reviewed literature which included fMRI studies as well as studies including neurotransmitter receptor function. Kaye et al. [10] also reviewed the neurobiology of AN, with a specific focus on fMRI studies related to appetite, reward and executive control. Owing to the focus on a specific topic of interest, however, prior reviews do not provide an exhaustive overview of the available fMRI research within the field of AN. Notably, a systematic review of functional neuroimaging of studies published between 1950–2009 provided a summary of the possible role of neurobiological factors in AN [11]. Since then, however, numerous fMRI studies on patients with AN have been published, and there is a need for an updated review that includes all experimental paradigms.

Given a rapidly developing and broad literature base, a scoping review may be a beneficial approach to provide an overview of the extent, range, and nature of research activity related to fMRI in AN. Scoping reviews have become an increasingly popular approach to mapping out the existing literature in terms of the volume, nature, and characteristics, especially when research questions are complex or heterogeneous [12, 13]. Scoping reviews tend to address broader topics in which many different study designs or experimental paradigms are applicable [12]. In contrast to a systematic review, which summarizes the best available research on a specific research question, a scoping review is a useful way to collect and organize important background information and develop an overall picture of the existing literature [14]. Because previous reviews on fMRI research in AN have typically focused on specific research questions or theoretical models, there is currently no up-to-date overview of research activity within this field. This would be beneficial in order to identify gaps in the literature and provide directions for both future research and systematic reviews. In the present study, we performed a scoping review of literature on fMRI research in AN published between 2010 and 2015.

## Main text

### Literature search

**Fig. 1:**
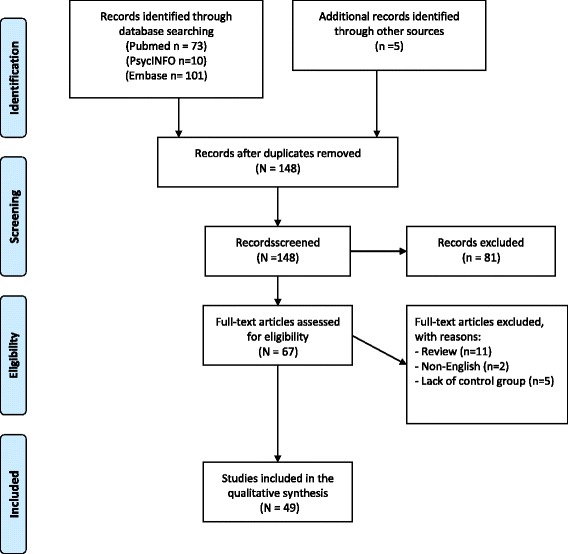
Flow of the search strategy

Relevant literature was identified via searches in PubMed, PsychInfo and Embase databases using the search terms “anorexia nervosa” AND “functional magnetic resonance imaging,” published between 2010 and 2015. In all, the main search resulted in 184 articles. A supplemental search was performed by manually-searching relevant journals and via Google Scholar. This supplemented our main search with five studies. After removal of duplicates, a total of 148 articles were assessed for eligibility. Studies were included if they were published between 2010–2015 in English language, used fMRI, and compared a healthy control group and a currently ill AN group, or a group of recovered individuals with AN (AN-REC) and a healthy control group. Additionally, only fMRI studies involving performance of tasks in the scanner were included, whereas connectivity and resting state studies were considered beyond the scope of the present review. Abstracts and titles were screened for relevance and eligibility, and 81 records were excluded. The full texts of the remaining 67 articles were examined in more detail. Eighteen studies were excluded for the following reasons: reviews (N = 11), non-English language (N = 2), or lack of control group (N = 5). In all, a total of 49 articles were included in the present study. Figure 1 illustrates a PRISMA flow chart of the search strategy (see Fig. 1).

### Study characteristics

The majority of studies included females only, with three exceptions [15–17]. Forty-four studies included an adult sample (age ≥18 years), whereas only six studies included an adolescent sample (age < 18 years). In the studies including ill patients, the mean body mass index (BMI) in all patient groups was <17.5. Seven studies included a patient group in which the mean BMI was ≤15. The majority of studies included patients diagnosed according to the Diagnostic and Statistical Manual of Mental Disorder- 4^th^ edition (DSM-IV) [18] criteria for AN, except for one study that used the fifth edition (DSM-5) [19]. Two studies did not specify DSM version. Fourteen studies included AN-REC patients.

In terms of sample size, the vast majority of studies had a sample of less than 20 patients, with one study sample of less than 10 patients. The largest sample size of the available studies was N =31.

### Experimental paradigms

Table 1 provides an overview of all studies, including experimental paradigms and main findings. The main findings represents significant differences between the experimental group (AN or AN-REC) and a healthy control group. A striking feature of the literature relates to the heterogeneity in experimental paradigms across studies. To provide an overview of these experimental paradigms, studies were organized according to the following ten categories: body and appearance (n = 10), neuropsychological functions (n = 9), food (n = 5), reward (n = 6), emotions (n = 5), taste (n = 5), pain (n = 3), social cognition (n = 3), compulsivity (n = 2), and self-identity (n = 1). These categories were chosen to illustrate the volume and focus of research activity over the past years, and to aid synthesis of findings within each domain of investigation. Where the paradigms overlapped across categories, studies were categorized according to the research purpose and main implications as stated by the respective authors. These topics are discussed below (in order of number of articles).

**Table 1:** Review of the literature on fMRI studies in AN (2010–2015)

Topic	Paradigm	Authors	Sample	DSM	BMI (SD) Age (SD)	Aim	Main findings
Body and appearance	View images of own and another woman’s body	Vocks et al., 2010 [20]	AN = 13 BN = 15 HC = 27	DSM-IV	15.8 (1.3) 29.1 (9.8)	Investigate neural correlates of looking at own and another woman’s body, and self-reported emotional response to the stimuli	Reduced activation in the left uncus, superior parietal lobule, medial frontal gyrus, fusiform gyrus, superior frontal gyrus and bilateral parahippocampal gyrus in response to own body. Increased activation in the amygdala in response to another woman’s body
	View images of a distorted body image, own and of another woman	Miyake et al., 2010 [22]	AN-R = 11 AN-BP = 11 BN = 11 HC = 11	DSM-IV	AN–R: 15.3 (1.8) 22.2 (4.1) AN-BP: 15.9 (1.9) 28.3 (4.5)	Investigate neural correlates of body image perception	Increased activation in the amygdala in both AN-R and AN-BP. Reduced activation in prefrontal cortex in AN –R patients
	View distorted images of own body	Mohr et al., 2010 [23]	AN = 16 HC = 16	DSM-IV	15.9 (1.3) 24.1 (3.4)	Investigate attitude towards the body and body size experience	Increased insula activity during processing of thin self-images
	View images of underweight, normal weight and overweight female bodies. Told to process images in a self-referring way	Fladung et al., 2010 [24]	AN = 14 HC = 14	DSM-IV	16.4 (1.8) 24.4 (7.6)	Examine the ventral striatal system in adults with AN	Increased activity in the ventral striatum to underweight stimuli
	View images of underweight, normal weight and overweight bodies, estimate body weight, and process stimuli in a self-referring way	Fladung et al., 2013 [25]	AN = 13 HC = 14	DSM-IV	16.6 (1.2) 16.0 (1.1)	Examine the ventral striatal system in adolescents with AN	Increased activity in the ventral striatum to underweight stimuli
	Viewing normal size and distorted pictures of own body	Castellini et al., 2013 [21]	AN = 18 HC = 19	DSM-IV	16.1 (1.4) 24.7 (7.6)	Explore neural network activated by processing of own body	Increased activity in the inferior frontal gyrus and middle temporal gyrus when viewing oversized images
	View images of idealized women, and compare with self	Friederich et al., 2010 [26]	AN = 17 HC = 18	DSM-IV	15.6 (1.4) 24.9 (5.6)	Investigate neural correlates of body dissatisfaction	Increased activation in the insula and premotor cortex, decreased activity of the rostral anterior cingulate cortex
	Negative words concerning body image	Miyake et al., 2010 [27]	AN-R = 11 AN-BP = 11 HC = 11	DSM-IV	AN-R: 15.2 (2.1) 27.0 (9.0) AN-BP: 15.5 (1.6) 27.2 (4.8)	Investigate functional abnormalities during processing of negative words concerning body image	Increased activity in the amygdala in both AN-R and AN-BP than in BN and controls, increased ventromedial prefrontal cortex in AN-BP and BN than in controls, and increased activity in the inferior parietal lobe in AN-R and AN-BP than controls
Body and appearance (cont.)	Viewing images of body checking	Suda et al., 2013 [28]	AN = 20 HC = 15	DSM-IV	15.7 (1.0) 27.0 (7.5)	Investigate brain activation to images of body checking	Reduced activation of the anteromedial prefrontal cortex and fusiform gyrus
	Viewing images of faces and houses at different spatial frequencies	Li et al., 2015 [30]	AN-REC = 15 HC = 15 BDD = 15	DSM-IV	20.4 (1.6) 23.6 (3.5)	Investigate visual and visuospatial processing in AN and body dysmorphic disorder (BDD)	Both groups had increased activation in secondary visual processing regions and dorsal visual stream
Neuropsych functions	Embedded figures test	Fonville et al., 2013 [31]	AN = 35 HC = 35	DSM-IV	16.0 (1.6) 23.0 (9.0)	Investigate neural patterns of activity in response to tests of central coherence	Reduced activity in the precuneus, increased activation in the fusiform gyrus
	Stop-signal task	Wierenga et al., 2014 [33]	AN = 11 HC = 12	DSM-IV	16.9 (1.5) 16.0 (2.0)	Explore inhibitory processing in AN	Inhibitory processing was related to reduced activity in dorsal anterior cingulate cortex, middle frontal gyrus, posterior cingulate cortex. Error processing was related to reduced activity in the middle frontal gyrus and posterior cingulate cortex
	Wisconsin card sorting test (WCST)	Sato et al., 2013 [35]	AN = 15 (AN-R = 9) (AN-BP = 6) HC = 15	DSM-IV	14.6 (1.5) 23.0 (7.0)	Evaluate brain activity in patients with AN while performing the WCST	Reduced activity in the ventrolateralprefrontal cortex and parahippocampal cortex during set shifting in all AN patients.
	Serial reaction time	Firk et al., 2015 [36]	AN = 19 HC = 20	DSM-IV	15.2 (1.5) 15.9 (1.5)	Examine implicit learning in AN	Reduced activity in the thalamus
	Letter n-back task	Lao-Kaim et al., 2014 [37]	AN = 31 HC = 31	DSM-IV	16.0 (1.6) 23.0^b^	Investigate the effect of increasing verbal working memory task difficulty on cortical functioning	No significant differences
	Working memory task, not emotional stimuli	Castro-Fornieles et al., 2010 [17]	AN = 14 (Male/female: 2/12) HC = 14	DSM-IV	14.9 (2.1) 15.0 (1.7)	Investigate brain activation during a non-emotional working memory task	Increased activation in superior parietal lobule and inferior temporal gyrus
	Go/no-go task	Kullmann et al., 2014 [34]	AN = 12 HC = 14 HCA = 12	DSM-IV	15.5 (1.5) 23.3 (4.7)	Test the hypothesis that neural correlates of behavioral inhibition are biased by the emotional information of the stimuli (food and physical activity), leading to different inhibitory patterns	Food stimuli were related to reduced activity in the putamen. Physical activity stimuli were related to increased activity in the prefrontal cortex and cerebellum
	Embedded figures test	Fonville et al., 2014 [32]	AN = 9 HC = 14	NA	15.9 (2.2) 22.0 (N/A)	Assess the effect of CRT on central coherence	A decreased task related activation in the fusiform gyrus and middle occipital gyrus
	Inhibition task	Oberndorfer et al., 2011	AN-R-REC = 12 HC = 12	DSM-IV	22.1 (19.0-25.4^a^) 29.4 (22-44^a^)	Investigate a prefrontal-cingulate network that is involved in inhibitory control	Less activation in the medial prefrontal cortex
Food	View images of high-calorie food and emotionally neutral images	Gizewski et al., 2010 [39]	AN-R = 12 HC = 12	DSM-IV	14.1 (1.8) 27.0 (18-52^a^)	Evaluate the influence of satiety, BMI and like/dislike ratings on cerebral activation patterns	Increased activation to food images in the dorsal posterior cingulate cortex in the state of hunger
	View images of food and non-food	Joos et al., 2011 [40]	AN-R = 11 HC = 11	DSM-IV	16.2 (1.2) 25.0 (5.0)	Clarify frontolimbic dysfunction in AN	Decreased activation in the posterior midcingulum. Increased right amygdala
	View images of food and non-food	Sanders et al., 2015 [42]	AN = 15 AN-REC = 14 HC = 15	DSM-IV	AN: 14.5 (1.7) 25.6 (5.0) AN-REC: 21.1 (1.9) 24.3 (5.0)	Test activation of bottom-up and top-down systems (model by Brooks et al. 2012)	In AN, increased activation in the cerebellum, middle frontal gyrus, and decreased activation in the precuneus and superior frontal gyrus. AN-REC showed increased activation in the caudate, cerebellum, middle frontal gyrus, and post central gyrus.
	View images of food and think about eating it	Brooks et al., 2012 [41]	AN = 18 (AN –R = 11) (AN-BP = 7) HC = 24	DSM-IV	15.7 (1.2) 26.0 (6.8)	Examine how cognitive systems interact with reward and appetitive systems in AN	Reduced activation in the cerebellar vermis, and increased activation in the visual cortex
	Anticipation task viewing images of food and object images	Oberndorfer et al., 2013 [43]	AN-REC = 14 HC = 12	DSM-IV	22.0 (1.6) 28.9 (6.6)	Determine whether AN-REC have abnormal anticipatory response to viewing pictures of food	Greater activation in the ventral anterior insula
Reward	Reward conditioning task	Frank et al., 2012 [9]	AN-R = 21 OB = 19 HC = 23	DSM-IV	16.1 (1.1) 22.5 (5.8)	Test whether they could find brain reward alterations in AN compared with individuals with normal or increased body weight	Increased activation in the anteroventral striatum, insula and prefrontal cortex compared with the HC and OB group
	Social reward Acceptance and rejection	Via et al., 2015 [45]	AN-R = 20 HC = 20	DSM-IV	16.9 (1.3) 28.4 (9.3)	Investigate brain responses to social reward (acceptance) and punishment (rejection)	Increased activation of dorsomedial prefrontal cortex during social acceptance and reduced activation in visual areas during social rejection
	Delay discounting task	Decker et al., 2015 [47]	AN = 30 HC = 22	DSM-V	16.8 (1.4) 19.3 (2.5)	Examine neural correlates of delay discounting in AN	Reduced activity in the dorsal anterior cingulate cortex and striatum
	Monetary guessing task	Bischoff-Grethe et al., 2013 [48]	AN-R = 10 HC = 12	DSM-IV	16.4 (1.4) 16.2 (1.8)	Replicate findings of altered reward and striatal response to reward and punishment	Increased activity in the striatum in response to losses compared to wins
	Monetary reward task	Ehrlich et al., 2015 [49]	AN-REC = 30 HC = 30	DSM-IV	21.2 (1.9) 22.0 (3.2)	Interrogate interactions between neural correlates of cognitive control and motivational processes in the reward system	Increased activation in the dorsolateral prefrontal cortex
Reward (cont.)	Delay discounting task	Decker et al., 2015 [47]	AN-REC = 23 HC = 17	DSM-IV	21.6 (0.3) 27.7 (1.6)	Investigate brain responses to rewards during hunger and satiated states to examine whether diminished response to reward could underlie food restriction in AN	Increased activation in the middle frontal gyrus.
Emotions	Implicit facial expression processing task (I-FEPT)	Fonville et al., 2014 [32]	AN = 31 HC = 31	DSM-IV	15.9 (1.6) 23 (10)^b^	Examine neural correlations of implicit emotion processing in AN	Increased activation in the fusiform gyrus
	Processing of negative words concerning interpersonal relationships	Miyake et al., 2012 [55]	AN = 30 HC = 20	DSM-IV	15.4 (1.7) 27.2 (6.5)	Investigate neurobiological relationship between alexithymia and AN	Increased activation in the superior temporal gyrus
	Implicit emotion processing task	Phillipou et al., 2015 [56]	AN = 24 HC = 25	DSM-IV	16.5 (1.1) 22.2 (5.5)	Investigate facial affect processing and the processing of own face through measures of emotion identification	Increased activation in the right inferior and middle temporal gyri and right lingual gyrus in response to own face
	Emotional conflict task	Bang et al., 2016 [57]	AN-REC = 22 HC = 21	DSM-IV	20.39 (1.66) 27.3 (5.14)	Explore neural responses to an emotional conflict task in recovered AN	Less activation in the bilateral amygdala, hippocampus and basal ganglia in response to emotional congruent stimuli
	View fearful and happy emotional faces	Cowdrey et al. 2012 [58]	AN-REC = 16 HC = 16	DSM-IV	21.3 (2.2) 23.1 (3.6)	Investigate neural processing of emotional faces in AN-REC	No significant differences
Taste	Intake of water and chocolate milk	Vocks et al., 2011 [51]	AN-R = 12 HC = 12	DSM-IV	14.1 (1.8) 27.4 (10.6)	Examine possible alterations in neural correlates of gustatory processing of food stimuli, and to test the impact of hunger and satiety	Hungry state was related to increased activation in the right amygdale and left medial temporal gyrus, and reduced activation in the right medial frontal gyrus
	Glucose intake	Van Opstal et al., 2015 [50]	AN = 10 HC = 11	DSM-IV	15.6 (1.0) 22.1 (3.3)	Elucidate hypothalamic functioning and structure in AN	No difference in hypothalamic activation
	Taste of sucrose (caloric) and sucralose (non-caloric)	Wagner et al., 2015 [53]	AN-REC = 14 BN-REC = 15 HC = 13	DSM-IV	20.9 (2.8) 26.4 (5.4)	Determine whether sensitization effects might underlie pathologic eating behavior when a taste stimulus is presented repeatedly	AN-REC showed a decreased sensitization to sucrose (caloric) and increased sensitization to sucralose (non-caloric) stimuli in the lentiform nucleus and thalamus
	Taste of sucrose and sucralose	Oberndorfer et al., 2013 [43]	AN-REC = 14 BN-REC = 14 HC = 14	DSM-IV	21.5 (2.8) 27.3 (1.4)	Interrogate gustatory neurocircuitry involving the anterior insula and related regions that modulate sensory-interoceptive-reward signals in response to palatable foods	AN-REC compared to controls had diminished response to tastes of sucrose in the anterior insula
Taste (cont.)	High-fat cream stimulus, water and non-caloric viscous stimulus	Radeloff et al., 2014 [52]	AN-REC = 15 BN-REC = 14 HC = 18	DSM-IV	21.0 (2.4) 25.2 (4.0)	To compare responses to a high-fat cream stimulus, water, and a non-caloric viscous stimulus in AN-REC and BN-REC and HC	The BN group showed increased activation in the anterior ventral striatum, however there were no differences in this region between the AN and HC group.
Pain	Heat pain thresholds, and thermal painful stimuli	Bär et al., 2013 [15]	AN-R = 19 HC = 19 Male/female: 3/16	DSM-IV	16.9 (1.2) 22.6 (5.3)	Test the hypothesis that altered processing of pain in the insula might account for reduced perception of pain	Reduced activity in the insula, cerebellum and pons
	Thermal pain stimulation	Bär et al., 2015 [16]	AN = 26 HC = 26 Male/female: 3/23	DSM-IV	17.0 (1.5) 22.9 (5.0)	Investigate neural correlates of body perception deficit	Increased activation in the right Brodmann area 23/31, left dorsal posterior and midcingulate cortex
	Painful heat stimuli	Strigo et al., 2013 [60]	AN-REC = 12 HC = 10	DSM-IV	21.9 (1.7) 29.7 (6.8)	Assess neural substrates of pain anticipation and processing	Greater activation in right anterior insula, dorsolateral prefrontal cortex and cingulate during pain anticipation. Greater activation in dorsolateral prefrontal cortex and decreased activation in the posterior insula during painful stimulation
Social cognition	Short videos of moving shapes	McAdams & Krawczyk, 2011 [62]	AN-REC = 17 HC = 17	DSM-IV	23.4 (4.2) 26.2 (7.0)	Examine neural correlates relating to thinking about social relationships	Reduced activation in the right temporoparietal junction
	Multiround trust game	McAdams et al., 2015 [63]	AN = 23 AN-REC = 19 HC = 21	DSM-IV	AN: 18.0 (1.5) 26.3 (8.1) AN-REC: 22.8 (2.7) 29.6 (8.3)	Compare neural responses in a social relationship	Diminished response in the precuneus and angular gyrus to benevolence (improved relationship) in both ill and recovered patients. In response to malevolence (deteriorating relationship), differed in the fusiform gyrus in ill patients only).
	Theory of mind task	Schulte-Rüther et al., 2012 [61]	AN = 19 HC = 21	DSM-IV	15.3 (1.5) 15.7 (1.5)	Identify neural mechanisms behind theory of mind deficits in AN	Reduced activation in the middle anterior temporal cortex and the medial prefrontal cortex. Increased activation in the medial prefrontal cortex
Compulsivity	Cue-reactivity	Rothemund et al., 2011 [65]	AN = 12 HC = 12	NA	13.6 (1.2) 24.0 (6.1)	Investigate compulsivity	Increased activation in caudate and precuneus
	Symmetry/order images e.g. uneven/chaotic/messy environments	Suda et al., 2014 [66]	AN = 22 HC = 24	DSM-IV	15.3 (1.1) 26.8 (8.0)	Examine brain activation in women with AN and HC during the provocation of symmetry/ordering-related anxiety	Reduced activation in the right parietal lobe (incl.precuneus) and the right prefrontal cortex in response to provocation. Inversely correlated with severity of symmetry/ordering symptoms
Self-identity	Identity appraisal tasks	McAdams & Krawczyk, 2014 [67]	AN-REC = 18 HC = 18	DSM-IV	19.8 (1.6) 26.1 (6.8)	Test the hypothesis that AN-REC show altered neural response while thinking about their identity	Reduced activity in the precuneus, dorsal anterior cingulate, middle frontal gyrus

BMI and age is reported for patients with AN. AN, anorexia nervosa; HC, healthy controls; BN, bulimia nervosa; AN-R, anorexia nervosa restrictive subtype; AN – BP, anorexia nervosa binge purge subtype; AN-REC, recovered anorexia nervosa; BN-REC, recovered bulimia nervosa; BMI, body mass index; CRT, cognitive remediation therapy; OB: obese; NA, not applicable. The main findings represents significant differences in the experimental group (AN or AN-REC) as compared to a healthy control group

^a^The values represents range

^b^The values represents median

#### Body and appearance

Ten fMRI studies have investigated neural activation related to the visual processing of body and appearance, both one’s own and another individual’s body. Upon viewing images of their own bodies, one study found that individuals with AN showed reduced activation in areas related to the attention network, and the authors suggested that this could indicate body-related avoidance behavior [20]. Distorted oversized images of own bodies has been associated with increased activation in the inferior frontal gyrus and middle temporal gyrus [21], as well as increased amygdala and reduced prefrontal cortex activation [22]. Other studies have reported increased activity in the insula [23] and ventral striatum [24, 25] in response to thin bodies. Comparing oneself to idealized bodies was associated with an increased activity in the insula and premotor cortex in patients, accompanied by reduced activity in the rostral anterior cingulate cortex [26]. Negative words related to body image elicited increased amygdala, medial prefrontal cortex and inferior parietal lobe activation [27]. Finally, one study found reduced activation of the prefrontal cortex and fusiform gyrus to images of body checking behavior [28]. Body checking involves repetitive checking of body shape and weight, and is a common feature of AN [29]. The only study investigating appearance-related stimuli in AN-REC individuals was conducted by Li et al. [30], who found increased activation in the secondary visual processing regions and the dorsal visual stream during passive viewing of faces. No other studies of AN-REC were identified using paradigms related to body or appearance.

#### Neuropsychological tasks

Neuropsychological tasks are standardized measures of cognitive functioning across various domains, and nine fMRI studies have employed neuropsychological testing in AN samples. Performance on a central coherence task was associated with reduced activity in the precuneus and increased activation in the fusiform gyrus in one study [31] and reduced task-related activation in the fusiform gyrus and the middle occipital gyrus in another study [32]. Inhibitory processing was associated with reduced activity in the dorsal anterior cingulate cortex, middle frontal gyrus and posterior cingulate cortex [33], and activation patterns were found to be dependent on the type of stimuli used in the task [34]. Performance on a set-shifting task was linked to reduced activation in the prefrontal cortex and parahippocampal cortex [35] whereas implicit learning was linked to reduced activity in the thalamus [36]. One study of working memory found increased activation in the superior parietal lobule and inferior temporal gyrus [17] while another found no differences between patients and controls [37]. One study investigated AN-REC individuals during an inhibition task, and found reduced activation in the medial prefrontal cortex [38].

#### Food

Neural responses to food stimuli have been investigated in five studies with mixed paradigms and results. Passive viewing of food images elicited increased activation in the dorsal posterior cingulate cortex and the insula in one study [39], and increased amygdala activity accompanied by reduced activity of the posterior midcingulate cortex in another study [40]. The authors suggested that results could be related to a dysfunction of top-down processes of the dorsal stream of emotion processing, however the evidence is not conclusive. When viewing food and told to think about eating it, patients with AN showed reduced activation in the cerebellum and increased activation in the visual cortex, suggesting a possible cognitive bias towards food stimuli [41]. One study comparing response to food images in ill and recovered AN patients found increased activation in cerebellum and middle frontal gyrus and decreased activation in precuneus and superior frontal gyrus in AN, while AN-REC showed increased activation in the caudate, cerebellum, middle frontal gyrus and post central gyrus [42]. One study also found that AN-REC showed greater activation than healthy controls in the ventral anterior insula to anticipation of viewing images of food [43]. These studies on AN-REC show that differences in processing of food stimuli are present not only during the underweight phase of AN, but show different patterns than during the illness phase.

#### Reward system

Food restriction and the pursuit for thinness in AN has previously been linked to the reward system [44], and six studies have investigated the neural correlates of reward in AN. In a social reward paradigm, social acceptance was related to decreased activation in the dorsomedial prefrontal cortex, and social rejections were related to increased activation in the secondary visual cortex [45]. Studies using monetary reward found altered activity in cortico-striatal reward systems in response to unexpected rewards [46] and during a delay discounting task [47]. In an adolescent AN sample, patients showed an exaggerated striatal response to losses compared to wins, which could be related to sensitivity to punishment typically seen in this patient group [48]. In AN-REC, a monetary reward task was associated with increased activation in the dorsolateral prefrontal cortex [49], whereas a delay discounting task revealed increased activation in the middle frontal gyrus [47].

#### Taste

Five studies have investigated the neural activation to gustatory processing of food stimuli or taste with mixed results. Despite the role of the hypothalamus in food intake processes and energy homeostasis, one study found no differences in hypothalamus activity during glucose intake in AN [50]. Another study found increased amygdala activation in AN patients when feeling hungry, consistent with heightened fear or emotional response to taste stimuli [51]. Results have also been mixed for AN-REC with some studies finding no differences in taste processing [52] but some have shown reduced sucrose sensitization [53] and reduced insula response to sucrose taste in this group [54].

#### Emotions

Five studies employed emotional paradigms involving affective facial stimuli or words. Negative words concerning interpersonal relationships were associated with increased activation in the superior temporal gyrus in AN [55]. Patients with AN also showed increased activation in the fusiform gyrus in an implicit facial expression task [32] and increased activity in right inferior and middle temporal gyri and right lingual gurys in response to own face stimuli [56]. In AN-REC, an emotional conflict task resulted in less activation in the bilateral amygdala, hippocampus and basal ganglia [57] while another study found no significant differences between AN-REC and healthy controls during viewing of fearful and happy faces [58].

#### Pain

Reduced perception of pain is a well-established phenomenon in AN [59], and three studies have investigated the neural correlates of pain in AN using thermal heat stimuli. AN patients showed reduced activity in the insula, cerebellum and pons [15] in addition to increased activation in the posterior, dorsal and medial cingulate cortex [16]. In AN-REC, anticipation of pain was associated with increased activation in the anterior insula, dorsolateral prefrontal cortex and cingulate, while receiving painful stimulation was associated with greater activation in the dorsolateral prefrontal cortex, and reduced activation in posterior insula [60].

#### Social cognition

Three studies have investigated aspects of social cognition. Two of these have focused on theory of mind, defined as the metacognitive ability to understand mental states of other people, such as beliefs, wishes and desires. Both currently ill [61] and recovered AN patients [62] showed reduced activation during a theory of mind task in areas in the temporal cortex and medial prefrontal cortex. In a trust game targeting social relationships, both ill AN patients and AN-REC showed reduced activation in the precuneus and angular gyrus in response to benevolence (improved relationships) [63]. Only the ill AN group showed differences in the fusiform gyrus in response to malevolence (deteriorating relationships).

#### Compulsivity

Two studies have used paradigms targeting compulsivity. Clinical features of AN comprise restricted eating, a compulsive pursuit of thinness, compulsive exercising and a relentless perfectionism [64]. During a cue-reactivity paradigm measuring compulsivity, patients with AN had increased activation in the caudate and precuneus [65]. Another study found that images provoking concern for symmetry and exactness and was associated with reduced activation in the superior frontal gyrus and the precuneus in AN patients [66].

#### Self-identity

One study has investigated self-identity, using an identity appraisal task [67]. In AN-REC, reduced activity in the precuneus, dorsal anterior cingulate and middle frontal gyrus was seen during this task.

## Conclusions

In the present study, we performed a scoping review of literature on fMRI in AN published between 2010 and 2015. We aimed to provide an update and overview of recent research activity related to fMRI studies in AN, regardless of experimental paradigm, to offer a comprehensive overview of this rapidly developing area of study. Given the diversity in experimental stimuli and paradigms applied, a scoping review offers a unique and beneficial contribution to our knowledge of research on fMRI approaches in this patient population. A total of 49 studies were included in the present review, which were categorized according to type of paradigm utilized. Specifically, these paradigms involved body-related stimuli (own and others), neuropsychological tests, food related stimuli, reward, emotions, taste, pain stimulation, social cognition, compulsivity, and self-identity. However, within each category there was a wide range of paradigms used, rendering an overall synthesis and direct comparison across studies complicated. It is worth noting that disorder-specific categories were overrepresented. Future research using non-disorder specific experimental paradigms would be beneficial to separate neural alterations associated with symptom provocation compared with general cognitive or emotional processing.

Collectively, main findings from these studies indicate altered neural activity across the brain, including the frontal, parietal, temporal and occipital lobes, as well as subcortical structures such as the amygdala, striatum, thalamus and the cerebellum. It is likely that alterations in multiple parts of the brain may jointly contribute to the array of symptoms and behaviors associated with the illness. It has previously been theorized that altered activation in regions related to the fronto-striato and limbic circuits have an important role in the pathophysiology of AN [44, 68–71]. Reduced activation in bottom-up regions such as the striatum, amygdala, hippocampus and cerebellum is often accompanied by increased activation in top-down regions such as the prefrontal cortex, dorsolateral prefrontal cortex, medial prefrontal cortex and the anterior cingulate cortex [72]. Similarly, Kaye et al. [44] have suggested that limbic information processing is strongly inhibited by inputs from cognitive domains such as the dorsolateral prefrontal cortex and the parietal cortex, suggesting that this dysregulation of neural activation could explain restrictive eating seen in AN. Although results from fMRI studies vary, recent findings are interesting in light of theories suggesting these neurocircuit dysfunctions in AN. Future studies would benefit from using well-established paradigms targeting these implicated neurocircuits (e.g. presentation of food images, taste stimulus, executive function tasks, decision making tasks, and reward tasks) to allow for direct comparison and evaluation of activation patterns between AN patients, AN-REC and individuals from other populations.

There are several noteworthy gaps and methodological considerations of the existing literature which we have identified. First, the vast majority of studies included less than 20 participants per condition. As argued by Mumford [73], power analyses are uncommon in fMRI studies, but conducting a power analysis can reduce the likelihood of underpowered fMRI studies, as well as the chances for type II errors. In addition, there are numerous confounds and pitfalls which characterize research in fMRI, ranging from data acquisition, data analysis and interpretation of results (for further details, see [74]). We identified only three studies which included males suffering from AN, thereby restricting the generalizability of findings to females alone.

Only six of the fifty studies in this review included an adolescent sample. Yet, importantly, neurobiological factors that are related to the etiology of the disorder may differ from neurobiological factors which sustain the disorder. Further research on adolescent samples is warranted to identify neurobiological factors involved in the onset and development of AN, as younger individuals with a shorter duration of illness might be distinguishable in fMRI results from older individuals with a chronic or protracted illness. Future systematic reviews could also be beneficial to investigate whether there are any distinctions between the findings from research on adult and adolescent samples.

The scope of our review entailed studies of currently ill, underweight patients, and collectively, this body of research cannot speak directly to whether brain alterations are trait- or state effects owing to the effects of malnutrition or starvation. Investigations of weight-recovered individuals offer a complementary line of research, and partly address the potential confounding effect of severe underweight. Findings from these studies demonstrate that neural processes are altered even in individuals that have recovered from AN. However, scarring effects in the brain may persist due to the chronic effects of malnutrition and underweight. Follow-up studies with long-term recovered individuals with AN might reduce the impact of scarring effects. Further exploration of neural processing associated with disorder specific (e.g. food or body stimuli) versus non-specific (e.g. neuropsychological tasks) in AN-REC individuals would be an interesting topic for future research. At the time of the current review, no studies have investigated the neural activity associated with viewing images of bodies of different size and weight in AN-REC. This could provide insight into how these processes are affected during recovery, and whether sensitivity towards specific body shapes persists over time. In our review, we found only four studies which classified subgroups of AN patients according to AN-restricting type and AN-binge/purge type, which may risk overlooking important distinctions along behavioral lines. With one exception, these studies applied DSM-IV [18] diagnostic criteria, and future research is warranted to replicate and extend findings to samples defined by DSM-5 [19]. Future studies are also recommended to investigate whether neural alterations commonly seen in AN are specific to AN, or whether these findings could be related to common underlying, or transdiagnostic, neurobiological processes seen in other mental disorders, such as anxiety disorders and depression. FMRI studies including individuals with AN as well as another psychiatric comparison group can shed light on the specificity of neural alterations. This could also help shed light on whether the alterations found in AN samples reflect actual dysfunctions relevant for clinical features. Clinically speaking, findings from neuroimaging studies have the potential to enrich our understanding of brain alterations in AN, and may lead to the development of theoretical models for AN.

A scoping review is beneficial for broadly mapping a field of research, and to identify gaps in research and practice. Yet, and as opposed to a systematic review, however, no formal assessment of the methodological quality of the included studies is typically performed [75]. The present review offers an updated picture of research activity on fMRI and AN, focusing on studies published during the past 5 years. Utilizing a scoping review methodology, we have identified gaps in the literature which are important to address to advance our understanding of AN, and characterized the range and diversity of experimental paradigms applied in fMRI studies for AN. This review also provides a summary of main findings, which collectively indicate altered activation in regions related to the fronto-striato and the limbic circuits.

## Abbreviations

AN: Anorexia nervosa
BMI: Body mass index
DSM-5: Diagnostic and statistical manual of mental disorders, Fifth Edition
DSM-IV: Diagnostic and statistical manual of mental disorders, 4^th^ Edition
fMRI: functional magnetic resonance imaging
I-FEPT: Implicit facial expression processing task
MRI: Magnetic resonance imaging
